# Structural basis for DNA strand separation by a hexameric replicative helicase

**DOI:** 10.1093/nar/gkv778

**Published:** 2015-08-03

**Authors:** Yuriy Chaban, Jonathan A. Stead, Ksenia Ryzhenkova, Fiona Whelan, Ekaterina P. Lamber, Alfred Antson, Cyril M. Sanders, Elena V. Orlova

**Affiliations:** 1 Department of Biological Sciences, Birkbeck College, Institute of Structural and Molecular Biology, Malet Street, London WC1E 7HX, UK; 2 Academic Unit of Molecular Oncology, University of Sheffield Medical School, Beech Hill Road, Sheffield S10 2RX, UK; 3 Departament of Biolody, University of York, York YO10 5DD, UK

## Abstract

Hexameric helicases are processive DNA unwinding machines but how they engage with a replication fork during unwinding is unknown. Using electron microscopy and single particle analysis we determined structures of the intact hexameric helicase E1 from papillomavirus and two complexes of E1 bound to a DNA replication fork end-labelled with protein tags. By labelling a DNA replication fork with streptavidin (dsDNA end) and Fab (5′ ssDNA) we located the positions of these labels on the helicase surface, showing that at least 10 bp of dsDNA enter the E1 helicase via a side tunnel. In the currently accepted ‘steric exclusion’ model for dsDNA unwinding, the active 3′ ssDNA strand is pulled through a central tunnel of the helicase motor domain as the dsDNA strands are wedged apart outside the protein assembly. Our structural observations together with nuclease footprinting assays indicate otherwise: strand separation is taking place inside E1 in a chamber above the helicase domain and the 5′ passive ssDNA strands exits the assembly through a separate tunnel opposite to the dsDNA entry point. Our data therefore suggest an alternative to the current general model for DNA unwinding by hexameric helicases.

## INTRODUCTION

In DNA replication helicases work ahead of the polymerase, catalyzing base pair separation to generate single-stranded nucleic acids from double-stranded precursors (1,2). Hexameric replicative helicases form rings around DNA and this topology is likely to favour stable DNA substrate binding, permitting long stretches of DNA to be unwound. The eukaryotic hetero-hexameric MCM2–7 complex is a replicative helicase as well as part of the replication initiation machinery (3,4). Many aspects of the catalytic mechanism of Minichromosome maintenance (MCM) and other hexameric helicases in general remain poorly understood (5), although structures of homo-hexameric helicases and their complexes with single stranded nucleic acid segments are emerging (6–9). However, there is no information that would indicate how these helicases bind DNA at the replication fork junction (RFJ) and how this may influence DNA unwinding.

**Figure 1.**
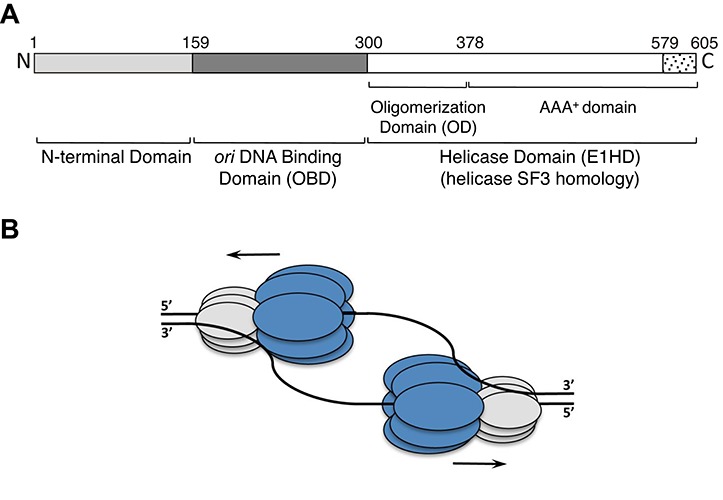
Schematic representation of the 605 amino acid BPV-1 E1 protein and helicase action. (**A**) The N-terminus (residues 1–300) contains the sequence-specific dsDNA *ori* binding domain (OBD) and an N-terminal regulatory domain. The C-terminal residues 301–605 form the helicase domain module (E1HD) comprising of the oligomerization domain (OD), AAA+ domain and acidic tail (residues 579–605). (**B**) Two hexameric helicases are thought to assemble at *ori* and drive bidirectional DNA replication. The helicase domain module of E1 is shown in dark grey (E1HD) and the N-terminal domains, including the OBD, in light grey.

The papillomaviruses (PV) are small dsDNA tumour viruses of significant medical importance (10) and the prototype of the group is bovine PV (BPV-1). PVs encode one highly conserved replication enzyme, E1, an initiator and helicase that is the viral equivalent of the MCM2–7 complex. The N-terminal half of BPV-1 E1 consists of a regulatory domain (residues ∼1–158) and a sequence specific origin of replication (*ori*) DNA binding domain (*ori* binding domain; OBD, residues ∼159–300), implicated in replication initiation (11). The C-terminal half (residues ∼301–605, the E1 helicase domain—E1HD) has helicase activity (12) and can be sub-divided into the oligomerization domain (OD, residues ∼308–378) and the AAA+ adenosine triphosphatase (ATPase) domain that includes a flexible C-terminal acidic tail (AT, residues 579–605) required for helicase processivity (7,13,14) (Figure 1). The superfamily 3 helicase E1 also shares significant sequence and structural homology with the initiator/helicase SV40 Large-T antigen (L-Tag) (15,16).

Crystal structures of the E1HD show hexamers with two tiers formed by the OD and the AAA+ domain. The latter bears the ATPase catalytic residues and the single-stranded DNA (ssDNA) binding hairpins that project into a central ssDNA binding tunnel. From these data models for nucleotide dependent translocation on ssDNA have been proposed (7,17), but the mechanism of base pair separation remains unknown. The E1HD structure with ssDNA bound in the central tunnel (7) fits with a ‘steric exclusion’ model for dsDNA unwinding, where active translocation on one ssDNA strand displaces the second ‘passive’ strand. Biochemical and structural studies support a similar mechanism in prokaryotic DnaB (18) and the dsRNA helicase Rho (8), among others. However, one important inference that can be drawn from the E1HD/ssDNA/ADP structure (7) and its revelation of the protein orientation relative to the direction of ssDNA translocation (3′–5′) (19) is that the N-terminal half of E1 (∼residues 1–300) must face the RFJ. Accordingly, the N-terminal part of E1 that includes the double-stranded DNA (dsDNA) binding OBD could influence base separation at the RFJ through interactions with the ss- and dsDNA (2). According to the currently accepted ‘steric exclusion’ mechanism, E1 moves in the 3′–5′ direction along the active ssDNA strand, with the N-terminal half pushing on the fork to separate dsDNA strands outside the E1 complex (20). However, the evidence for 5′-strand exclusion in this model is indirect and the role of individual E1 domains in unwinding remains ill defined. The lack of structural data on complexes with DNA replication fork substrates could be explained by the expected highly dynamic and mobile nature of such assemblies and by potential symmetry perturbations and conformational variation induced by binding of such DNA, hindering crystallization and high-resolution EM reconstruction. Indeed, high-resolution structural data for hexameric helicases in general are available only for complexes with short single-stranded nucleic acid segments bound (7,9), establishing that the active strand is pulled through the central tunnel of the C-terminal motor domain.

To advance understanding of the mechanism of dsDNA strand separation we have used electron microscopy (EM) and single particle analysis to obtain structures of the full length BPV helicase E1 and two complexes of E1 bound to a RFJ end-labelled with protein tags. We have used negative stain EM that allows imaging of samples with high contrast to accurately reveal positions of labels that are 20–60 kDa in size, as employed in this study. The combined approach of DNA end-tagging and negative stain EM indicated the dsDNA entrance and passive ssDNA strand exit points in the helicase–RFJ complex. Structural data correlate with DNA–protein contact points in RFJ DNA bound E1 complexes determined by nuclease footprinting. Notably, the data indicate that dsDNA enters the E1 complex through a tight side tunnel and not along the central axis of the hexamer as commonly assumed for E1 and other hexameric helicases (18,21–23), while the 5′ ‘passive’ strand exits the complex via a channel opposite the dsDNA entry tunnel. DNA unwinding is therefore taking place at the entrance to the helicase domain, inside and not outside the hexamer.

## MATERIALS AND METHODS

For the full experimental protocol, please see Supplementary Data.

E1 proteins were purified as previously described (12,24). The monomers for assembling the monovalent tetrameric streptavidin complex (MTS) were expressed from plasmids pET21a-Streptavidin-Dead and pET21a-Streptavidin-Alive (25), which were supplied by Dr Alice Ting's Laboratory, MIT, via Addgene.org. Helicase and OH• footprinting were performed as previously described (24,26), with minor modifications indicated in the supplementary experimental procedures. Protein–DNA complexes for EM were prepared by incubating protein and DNA at a 6:1 molar ratio before size exclusion chromatography (SEC) on a Superdex-200 (GE Healthcare) gel filtration column (10 mM Tris–Cl pH 8.0, 225 mM NaCl, 5% v/v glycerol, 2 mM dithiothreitol (DTT) , 0.1 mM phenylethanesulfonylfluoride (PMSF), 0.1 mM ethylenediaminetetraacetic acid).

Images were recorded using a Tecnai F20 electron microscope operated at 200 keV, images were recorded using a Gatan Ultrascan 4000 4k × 4k CCD camera at a nominal magnification of 62 000. Image processing was performed with CTFIT (27) and IMAGIC-5 (28,29), with the alignment and classification of images performed as previously described, and references therein. Angular orientations of class averages were determined by angular reconstitution. Three-dimensional (3D) maps were calculated using the exact-filter back projection algorithm (29,30) (see Supplementary Table S1). Interpretation and illustrations were done using Chimera (31).

## RESULTS

### The full-length E1 helicase preferentially unwinds forked DNA substrates

**Figure 2.**
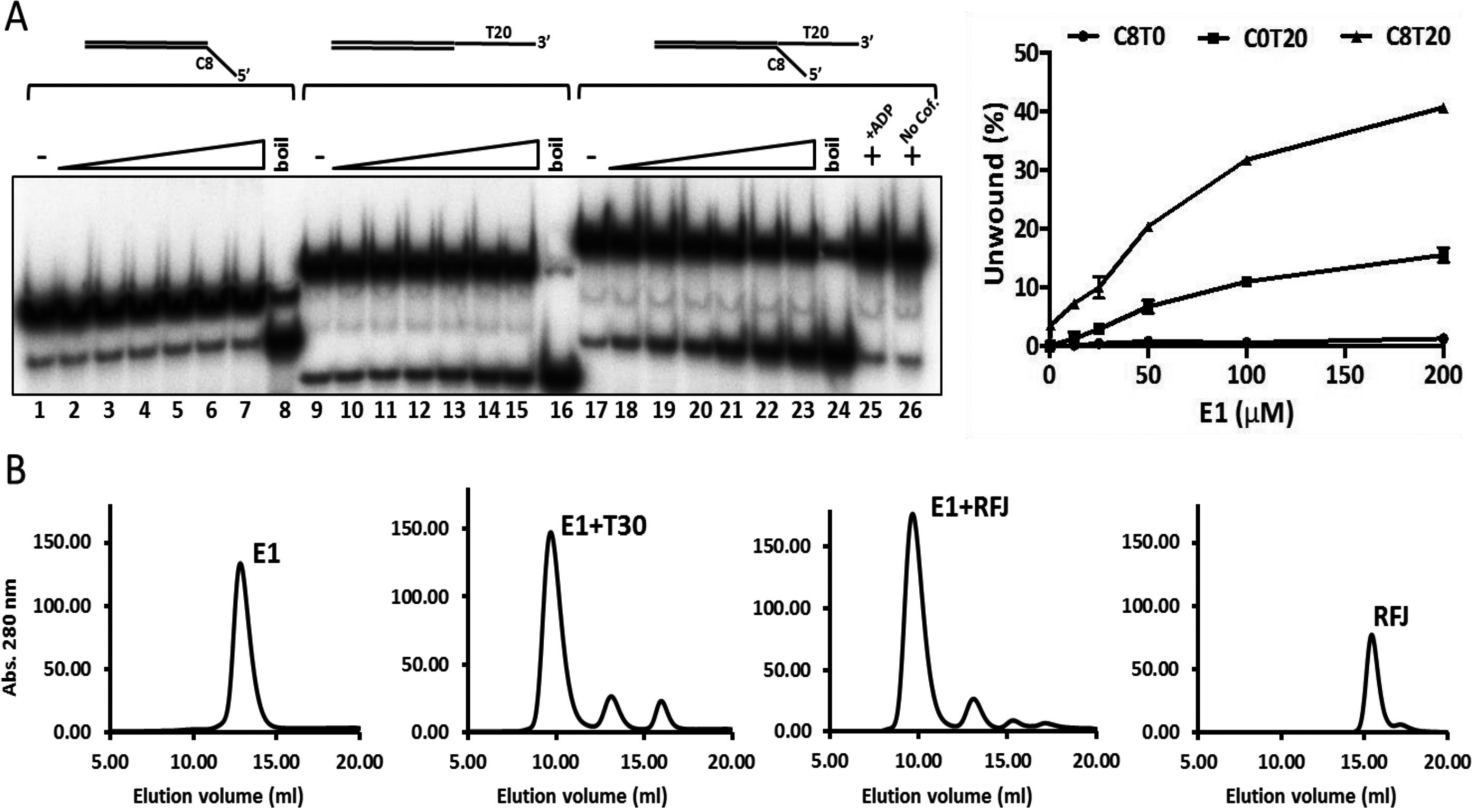
Helicase activity of E1 and protein–DNA complex formation. (**A**) Full length E1 preferentially unwinds forked test substrates with 5′ and 3′ ssDNA tails. The DNA substrate concentration used was 0.25 nM and the protein titration range was 6.25, 12.5, 25, 50, 100 and 200 nM E1. Heat denatured substrate is indicated as ‘boil’. No unwinding was observed in reactions containing ADP in place of ATP (lane 25) or without nucleotide cofactor (lane 26). The graph on the right shows the data from three independent repeats (mean and SD). (**B**) Formation and resolution of E1–DNA complexes by gel filtration chromatography. E1 (50 μM) and DNA substrates were mixed at a molar ratio of 6:1 and incubated at room temperature for 15 min before chromatography. Complex formation is shown for ssDNA T30 and the RFJ like substrate with 10 bp dsDNA, 3′ T20 and 5′ C10 ssDNA arms. Similar results were obtained with the forked substrate used in the helicase assays (not shown). All DNAs alone eluted late in the chromatogram as illustrated for the fork dsDNA 10, ssDNA 3′T20 and 5′C10 (‘RFJ’ trace on the right).

E1 is a 3′–5′ helicase that initiates unwinding of dsDNA substrates with 3′ ssDNA tails (19). To establish the basic configuration of a substrate appropriate for structural analysis we compared the unwinding of simple linear partially single- and double-stranded substrates to a forked substrate with 5′ and 3′ ssDNA tails using a radiometric strand displacement assay. In accord with previous observations, a short double stranded oligonucleotide substrate (30 bp dsDNA) with a 3′ T20 but not a 5′ C8 ssDNA tail was unwound by E1 (Figure 2A, lanes 1–8 compared to 9–15). However, the extent of unwinding of a forked-substrate with both 3′ T20 and 5′ C8 ssDNA tails increased approximately three-fold compared to the substrate with only a 3′ T20 tail (Figure 2A, lanes 17–24 compared to 9–16). Strand displacement was observed in the presence of ATP/Mg^2+^, but not in reactions without nucleotide cofactor or with adenosine diphosphate (ADP) in place of ATP (lanes 25 and 26).

### Formation of E1–DNA complexes

Like the E1 helicase domain residues 299–605 (12), purified full length E1 (E1FL) formed a stable complex with ssDNA (T30) in the absence of nucleotide cofactors and the peak fractions of the complex purified by gel-filtration chromatography showed a homogeneous population of hexameric particles when examined by negative stain EM (Supplementary Figure S1A and E). Using RFJ substrates with dsDNA arms from 10–30 bp and ssDNA arms 3′ T20 and 5′ C8–12, we observed similar stable complexes in gel filtration chromatography (complex formation with a substrate comprising of 10 bp dsDNA and 3′ T20 and 3′ C10 ssDNA arms is shown in Figure 2B). In all cases, the chromatograms did not indicate the presence of intermediate species eluting between the monomeric and hexameric populations that were effectively resolved by the chromatography.

### Electron microscopy and 3D reconstruction of E1 helicase complexes

**Figure 3.**
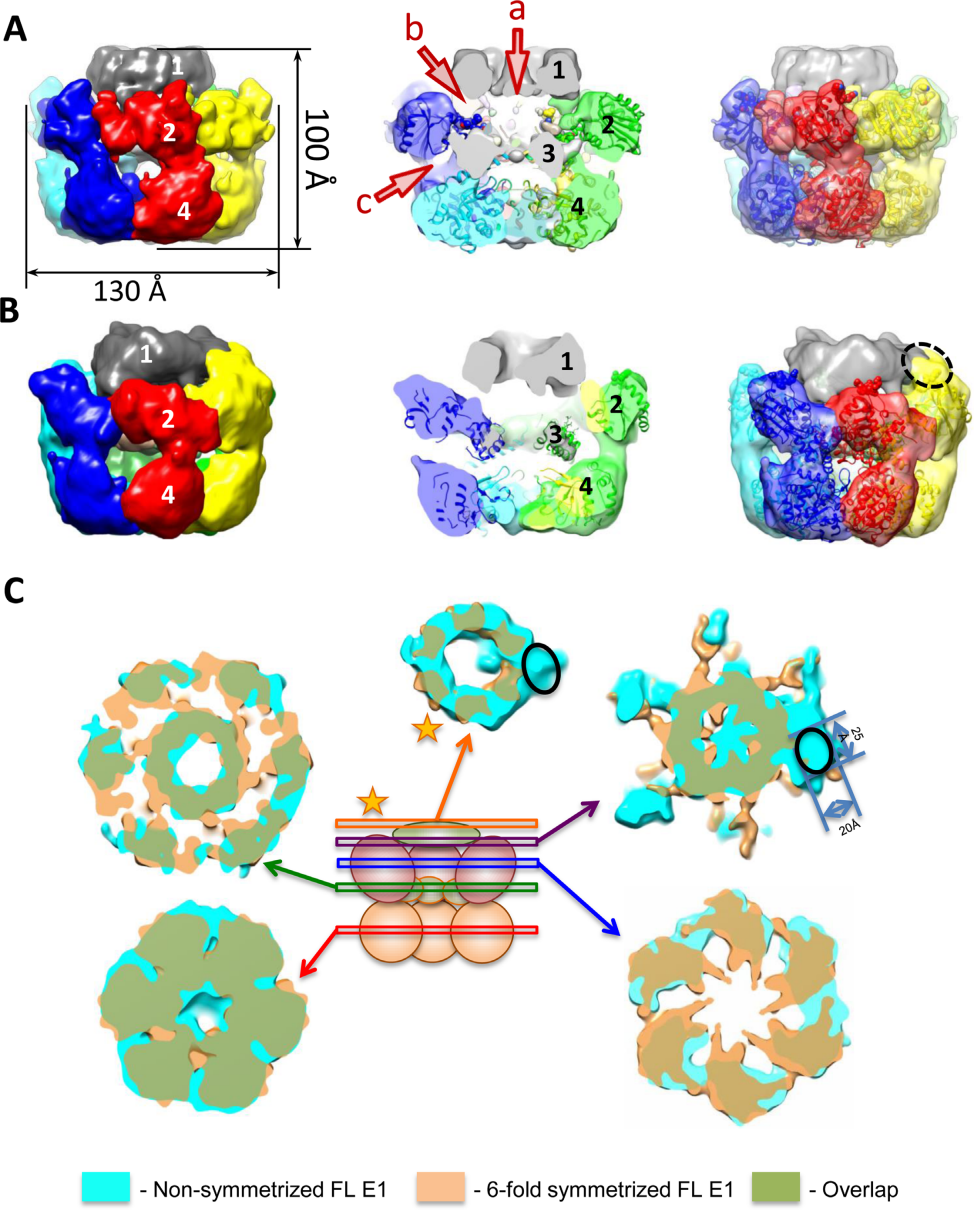
EM structures of full length E1 bound to ssDNA. The domains of E1FL are numbered: 1, N-terminal residues 1–158; 2, OBD; 3, OD and 4, AAA+ domain. Atomic structures of the E1HD (2V9P (17)) and the OBD (1KSX, (33)) are docked into the structures. (**A**) The symmetrized structure of E1FL–ssDNA. Arrowheads a, b and c indicate openings to tunnels within the E1FL structure. The opening to central tunnel ‘a’ is indicated at the N-terminus only. (**B**) The asymmetrical structure of E1FL–ssDNA. The same triple-tiered organization as in (A) was observed with six clear subunits in the bottom and middle rings. However, asymmetry was observed as the presence of a bulk of density on the outside of the upper ring, outlined by the blue ellipse. (**C**) Comparison of the symmetric and asymmetric reconstructions of the E1FL–ssDNA complex. Overlay of the symmetrized and asymmetrical structures. The upper tier corresponding to N-terminal domain 1 (indicated by a star) is located at the same height in both symmetrized and asymmetric structures. There are no distortions in that region except for the additional density on the outer side of the N-terminal tier in the asymmetric structure indicated by the black oval. Areas of overlap between the two structures are shown in green colour.

First, a structure of the purified E1FL–T30 complex examined by negative stain EM (Supplementary Figure S1A and E) was determined with C6 rotational symmetry imposed during the initial structure determination. Having this restraint we did not expect to reveal the position of the DNA relative to the individual subunits of the complex. The E1FL hexamer structure, determined at a resolution of 18 Å (Figure 3A), has a maximum diameter of 130 Å and a height of 100 Å and can accommodate ∼410 kDa of protein mass, in agreement with the predicted 409 kDa. The shape of the complex resembles a triple-tiered ring, with the middle ring having the larger diameter and the upper ring the smallest (Figure 3A, left). To determine the organization of the E1FL particle, as described further below, we used a comparison of the EM projections of E1HD (Supplementary Figure S2) and E1FL, and domain identification using antibody labelling. There is a central tunnel (‘a’) that varies in diameter along its length: at the bottom of the structure (C-terminus) the tunnel is ∼20 Å in diameter; travelling upwards the tunnel ‘a’ forms a chamber within the HD area, which expands to 27 Å below sub-domain 3 (Figure 3A, middle) and then it narrows to 23 Å in diameter at the entrance to the central tunnel formed by the upper ring. A second chamber is formed by the middle and upper rings and it is connected to the protein surface via twelve discernible tunnels (Figure 3A, middle and Supplementary Figure S2).

After obtaining the structure with symmetry C6 imposed we verified whether the structure had the same appearance without applying any symmetry during the reconstruction process. For the asymmetric reconstruction the same dataset was used and the initial model was obtained using mostly tilted views and a few end views of the complex. The structure demonstrated the same general features as described above for the symmetrized structure but was obtained at a lower resolution of ∼23 Å. We observed the same triple-tiered organization with six clearly defined subunits in the bottom and middle rings (compare Figure 3A–C). Strikingly however, in the upper ring there was clear asymmetry revealed by the presence of a bulk of density on the outer side of the upper ring (right panel, Figure 3B and C). The size of this bulk, highlighted with the black oval in Figure 3C, is about 20 × 25 Å. The centre of this bulk of density is located at a distance of ∼36 Å from the centre of the opening to central tunnel ‘a’. As expected, the lower resolution of the asymmetrical structure did not allow as clear definition of the inner channels of the complex compared to those defined in the symmetrical reconstruction and did not reveal any densities inside of the inner chamber of the E1 hexamer that could be reliably identified as ssDNA.

**Figure 4.**
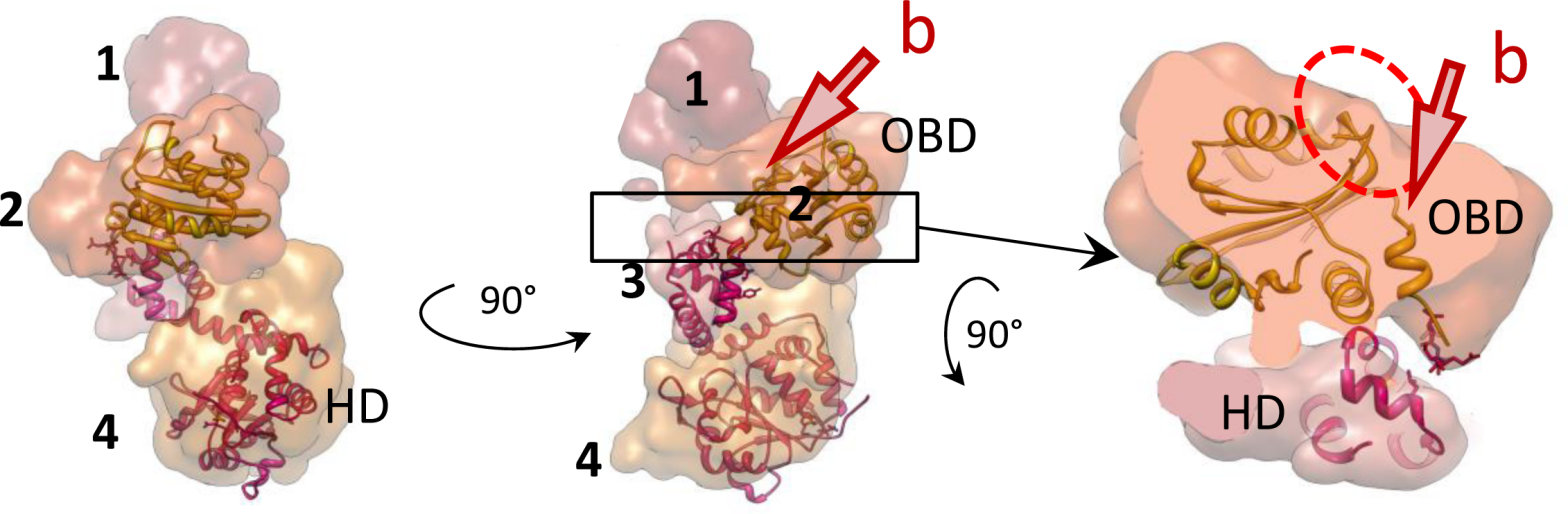
Structural organization of one E1 subunit. Atomic structures of E1HD (17) and the OBD (33) docked into one E1 subunit extracted from the hexamer. The domains are coloured in different shades and indicated with numbers as in Figure 3. Two views (left and centre) are of a single subunit rotated by 90° around the central (vertical) axis of the helicase. On the right, the segment indicated by the box in the central panel has been extracted and rotated 90° around the horizontal axis. The DNA binding segments of the OBD are circled in red and their corresponding positions in the E1FL hexamer are near the opening to the tunnel ‘b’.

Analysis of the symmetric and asymmetric structures (Figure 3) has allowed us to identify a single E1 subunit in the hexameric E1FL structure. A monomer, extracted using the segmentation function of the molecular modelling programme Chimera (31), is shown in Figure 4 to demonstrate the overall domain organization. Globular domain 4 shapes a dense ring at the base of the hexamer; domain 3 forms the inner ring that constrains the central tunnel. Domain 2 creates the outer rim of the wide middle ring of the structure. Finally, small domain 1 is located on top, forming the narrowest ring of the complex. Accordingly, the two sets of six side openings, ‘b’ and ‘c’ to corresponding tunnels observed in the E1FL hexamer (Figure 3A, middle) can be described as follows: tunnel ‘b’ is formed by E1 domains 1 and 2, has a length of ∼25 Å and a cross-section of ∼18 × 20 Å. Therefore, like the central axial tunnel ‘a’, it is wide enough to accommodate dsDNA. Tunnel ‘c’ is formed by domain 4 of one subunit and domains 2 and 4 of the neighbouring subunit and has a cross-section of ∼13 × 20 Å with a length of ∼30 Å. Being smaller than tunnel ‘b’ it could accommodate ss- but not dsDNA. Significantly, the additional bulk of density observed on the upper tier of the E1FL–ssDNA complex after asymmetrical reconstruction (Figure 3B, right panel and Figure 3C) coincides with a tunnel opening ‘b’.

E1 hexamers translocate 3′–5′ on ssDNA and this ‘active’ 3′ ssDNA strand, also corresponding to the leading replication strand in this case, is engaged by the ssDNA binding segments of the AAA^+^ domain (7,24). The E1FL–ssDNA structures, revealing previously unidentified tunnels and the additional asymmetrically positioned density in the upper tier (Figure 3), led us to determine directly the point of dsDNA entry into the complex and the path taken by the second lagging or ‘passive’ 5′ ssDNA strand of an engaged replication fork.

**Figure 5.**
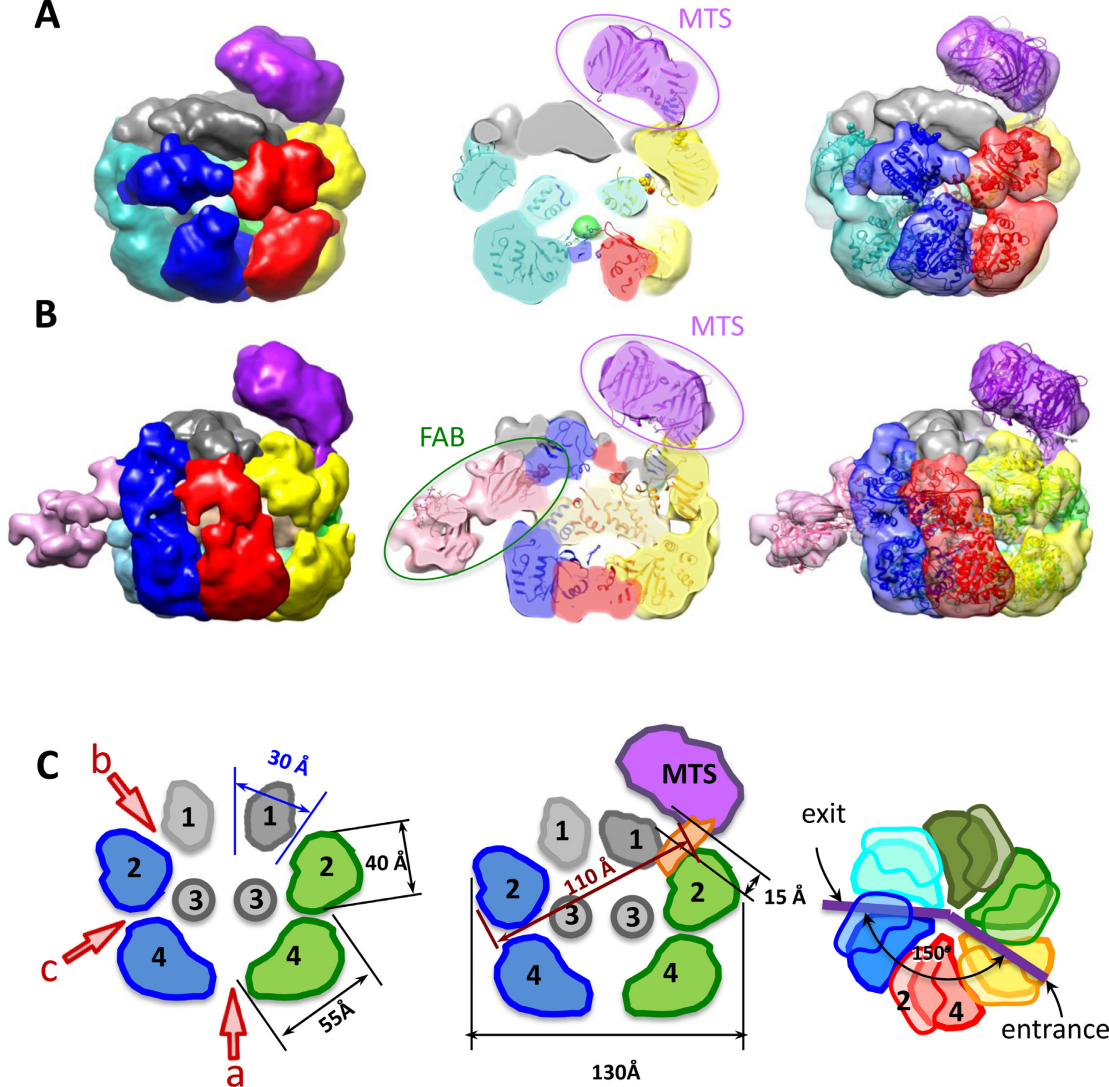
EM structures of E1FL hexamers bound to protein tagged RFJ DNA. (**A**) Structures of E1FL complexes with monovalent tetrameric streptavidin (MTS) bound to the 5′ end of the dsDNA arm. (**B**) The structure with anti-digoxigenin Fab bound to the 5′ ssDNA arm in addition to MTS bound to the dsDNA of the RFJ. Subunits of structure are shown in different colours. Side views and cutaway views are shown with atomic models of E1HD and the OBD docked into EM maps (central column and a column on the right). (**C**) Cartoon representations of the unlabelled and MTS labelled E1 structures with distances and angles between tunnel openings indicated. There is a 15 Å gap present between upper part of the hexamer and MTS.

First, we constructed a synthetic DNA replication fork with 30 bp dsDNA and 3′ T20 and 5′ C8 ssDNA tails where the 5′ end of the dsDNA was labelled with biotin. After formation of protein–DNA complexes, MTS (25) was bound and complexes were purified by gel filtration. Although sodium dodecyl sulphate-polyacrylamide gel electrophoresis analysis of the hexameric E1FL fractions clearly showed the specific incorporation of MTS, we failed to clearly visualize the binding of MTS in the hexameric particles examined by negative stain EM (not shown). Subsequently, we adopted a substrate with a shorter dsDNA sequence, 10 bp dsDNA and 3′ T20 and 5′ C10 ssDNA tails, labelled with biotin on the 5′ end of the dsDNA with or without digoxigenin on the 5′ ssDNA end. Hexameric E1FL complexes were first assembled on these substrates before MTS or anti-digoxigenin Fab were bound, as appropriate, and complexes purified by gel-filtration chromatography (Supplementary Figure S3). Purified complexes were then used for negative stain EM, single particles were aligned, classified and the 3D-structure of the complexes determined without imposing symmetry (Figure 5, Supplementary Figures S1 and S3). The final resolution of the structures obtained was 20 Å.

The structure of the E1FL–RFJ complex with the dsDNA end of the RFJ 5′-labelled with MTS has the same overall triple ring organization as described above. An additional density at the top of the complex located off the central axis of the hexamer was attributed to MTS (Figure 5A, Supplementary Figures S1C and S1G). Subsequently, we determined the structure of the double-labelled E1FL–RFJ complex with MTS on the dsDNA arm (5′-end) and anti-digoxigenin Fab on the 5′ ssDNA arm (‘passive’ strand) (Figure 5B, Supplementary Figure S1D and H). Only ∼30% of the E1Fl–RFJ complex were labelled with MTS and Fab, so sorting of the particle images using 3D statistical analysis was necessary to extract dual-labelled complexes (30). The structure of this complex revealed two additional external densities: one at the same position as in the complex with only the dsDNA labelled with MTS and the second extra mass located on the side of the complex, nearly opposite to MTS, at the level between the high density lower ring and middle wide ring (Figure 5B). Interestingly, both structures labelled with MTS on the dsDNA arm (without or with Fab on the 5′ ssDNA arm) were distorted, having the upper smallest ring pressed obliquely into the large ring of the OBD domains beneath the upper, extra, density (MTS). Overlay of these two structures demonstrated excellent overlapping of the density on the top of the complex, indicating unambiguously the position of MTS and its link to the E1 complex. The remaining extra density at the side of the dual-labelled complex was assigned to the Fab. In the structure, the Fab label disturbs the position of the OBD domains in the middle ring and appeared to be partially submerged into the complex. The distortions induced by MTS and Fab therefore demonstrate a degree of flexibility in the middle and upper tiers of E1 formed by domains 1 and 2.

### Analysis of the E1FL structures

The structural organization of the E1FL hexamers was determined stepwise using three approaches. First we used antibodies (ab) specific to residues 1–129 and negative stain EM coupled with statistical analysis to determine the position of the corresponding N-terminal segment in the E1FL complex, which was shown to correspond to domain 1 at the top of the complex (Figure 3 and Supplementary Figure S2A).

Second, we aligned the E1FL EM structure with a 19 Å resolution EM structure of E1HD (residues 299–605) that we obtained using similar methods to the E1FL structure (Figure 3 and Supplementary Figure S2B). A correlation analysis revealed that the best alignment was achieved with the bottom ring of the E1FL structure (cross correlation coefficient, CCC of 0.84).

Third, available atomic coordinates of E1 domains were docked into the EM maps (Figure 3A). Following the antibody labelling experiments and comparison with the EM projections of E1HD described above, an initial manual fitting of E1HD crystal structures (PDB IDs: 2GXA (7) and 2V9P (17)) in the lower ring (HD) was refined using Veda software (32) and Chimera (31). The fitting resulted in a CCC = 0.72, which was reduced to ∼0.2 for the upper tier of the complex indicating a better consistency between E1HD crystal structures and the lower tiers of the E1FL structure. An automated docking of the atomic structure of the OBD, residues 159–303 (PDB ID: 1KSX, (33)), into the E1FL map showed that the wide tier formed by domain 2 corresponded well to six OBD domains (Figure 3A, CCC = 0.76, Supplementary Table S2). Importantly, in this docked model the C-terminal linker connecting the E1 OBD and HD (amino acids 300–303) overlaps with the corresponding residues at the N-terminus of the E1HD X-ray structure fitted into the E1FL maps, confidently fixing one point of alignment. To further verify the assigned OBD orientation we inserted the HA-epitope sequence into a small surface loop (residues 225–228) after residue 226. The automated fitting of the OBD predicted that these residues would be exposed on the outer surface of E1FL. Again, antibody-labelling experiments demonstrated, in the presence of anti-HA antibodies, an additional bulk of density of the size and shape expected for IgG antibody (Supplementary Figures S5 and S6) at the position assigned to the OBD. Overall, the results of fitting the X-ray structure of the OBD into the EM maps were very similar for all the E1 complexes analysed, favouring the same orientation of the OBD (Supplementary Table S2). Interestingly, the location and orientation assigned to the OBD resulted in the dsDNA binding face of the OBD, circled with the dashed red line in Figure 4, being positioned at the outer surface of the E1FL complex, at the entrance into the side tunnel ‘b’.

### The footprint of E1FL bound to a RFJ DNA substrate

**Figure 6.**
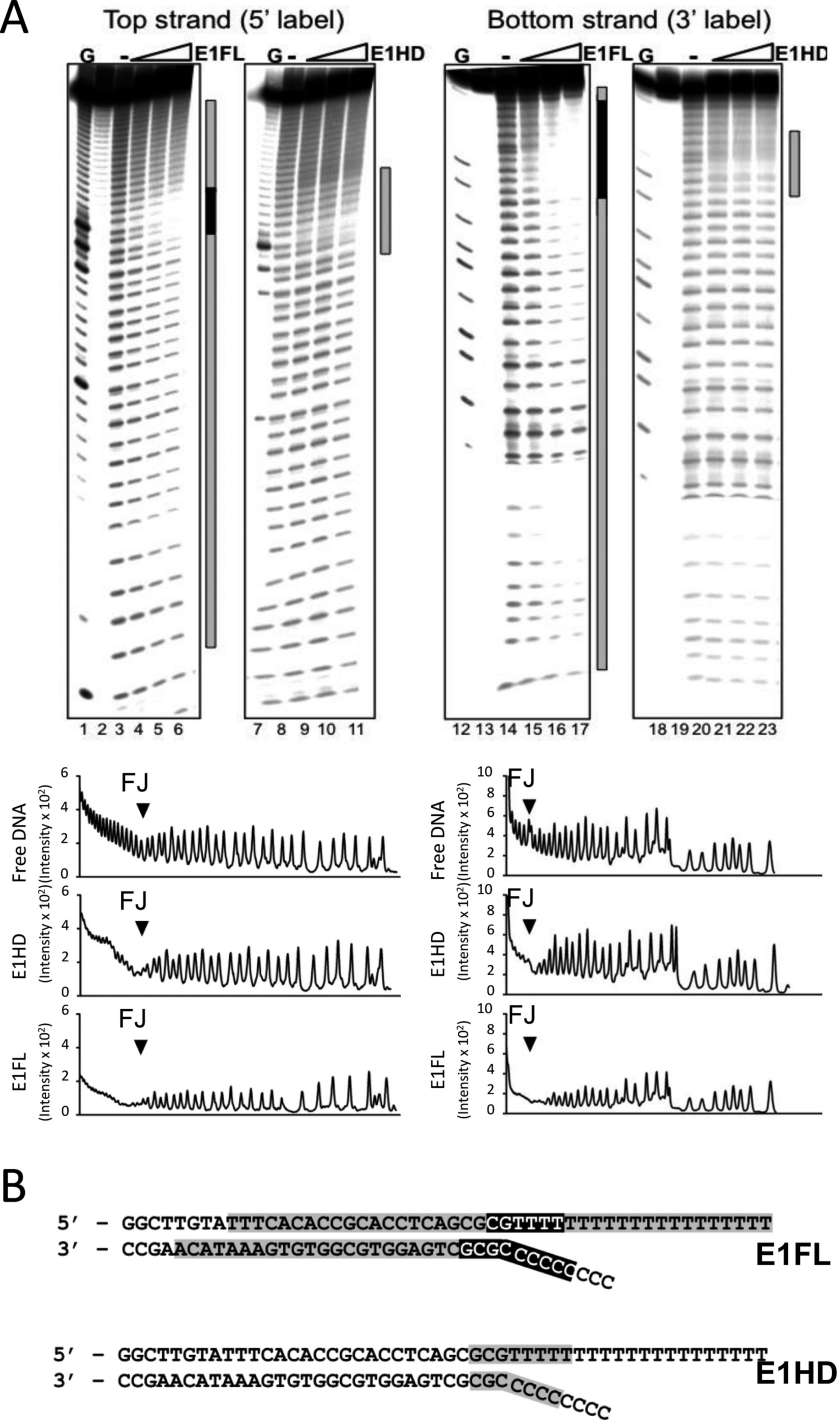
Hydroxyl radical footprinting of E1–RFJ complexes. (**A**) Lanes 1–11, ^32^P 5′-end labelled top strand and lanes 12–23 3′-end labelled bottom strand. Lanes 1, 7, 12, 18, G ladder. Lanes 2, 13 and 19 show unreacted substrate. The protection patterns were analysed using the lane-profiling tool of the phosphorimaging software (shown below the gel images). A peak height reduction of 80% or more was scored as complete protection (indicated in black in the annotations on the right of each gel image; partial protections are shown in grey). (**B**) Projection of the OH• footprinting pattern on the fork sequence.

Our structural analysis suggests that the E1 N-terminal domains 1 and 2 adopt a configuration that could accommodate the RFJ DNA. We tested this by comparing the hydroxyl radical (OH•) nuclease protection ‘footprints’ of E1FL and E1HD bound to a RFJ-like DNA molecule (Figure 6). The DNA binding substrate used was identical to the fork substrate used in the helicase assays shown in Figure 2A, with 30 bp dsDNA, T20 3′ active ssDNA strand and a C8 5′ passive ssDNA strand. The OH• is a small, diffusible, nuclease and DNA is protected from cleavage only where there are very tight contacts with protein (34). We anticipated that the OH• could diffuse into the protein complex through the well-defined openings and expected that only segments of RFJ-like DNA that interact tightly with protein would be protected.

Figure 6A lanes 4–6 show the footprint of the E1FL complex bound to the top strand of the DNA fork (5′ ^32^P-end labelled). Near complete protection of ∼6 nt at the fork junction (Figure 6B) indicates the presence of close contacts between E1FL and DNA. In addition, we observed a weaker and diminishing protection extending over ∼20 bp of dsDNA from the junction point and over the entire 3′ and 5′ ssDNA arms. Importantly, the protection pattern over the dsDNA lacks features such as periodic protection and susceptibility to cleavage observed for DNA lying on a protein surface (34) and is best interpreted as encirclement of the dsDNA in a protein sheath. In comparison, for the E1HD we observed only partial protection over about 8 nt on each DNA strand centred over the fork junction (lanes 9–11 and Figure 6B). Similar patterns of protection to the 5′-end labelled top strand were observed for the 3′-end labelled bottom strand for both E1FL and E1HD complexes, respectively (lanes 15–17 and 21–23). These data therefore indicate that the E1HD makes a limited set of weak protein–DNA interactions with the RFJ, while in the intact E1FL complex there is a more extensive set of interactions with all arms of the fork.

### dsDNA and the 5′ ssDNA strand enter and exit E1 through opposing side tunnels

The position of the MTS label attached to the dsDNA end of the DNA fork was identical in the single- and double-labelled E1 helicase particles. Figure 5A shows the structure of the complex labelled with MTS on the 5′ dsDNA end of the fork, docked with the atomic coordinates for E1 and MTS (PDB ID: 3RY1, (35)). Figure 5B shows the structure of the double-labelled E1FL complex and the fitting of the atomic coordinates for E1, MTS and the anti-digoxigenin Fab X-ray models (PDB ID: 1IGJ, (36)). In each case, the electron density maps show that the position of the MTS is not on the top of the central tunnel. Moreover, there is a significant gap (∼15 Å) present between the upper part of the hexamer and MTS makes contact with the E1 surface at a point that is ∼45 Å away on the upper side of the complex (Figure 5C and Supplementary movie S1). The MTS contact with E1 is in the vicinity of the dsDNA-binding site of the OBD at the entrance into a side tunnel ‘b’. A clear region of electron density connects the MTS with the entrance to tunnel ‘b’, while the opening to the central tunnel ‘a’ is unobstructed.

**Figure 7.**
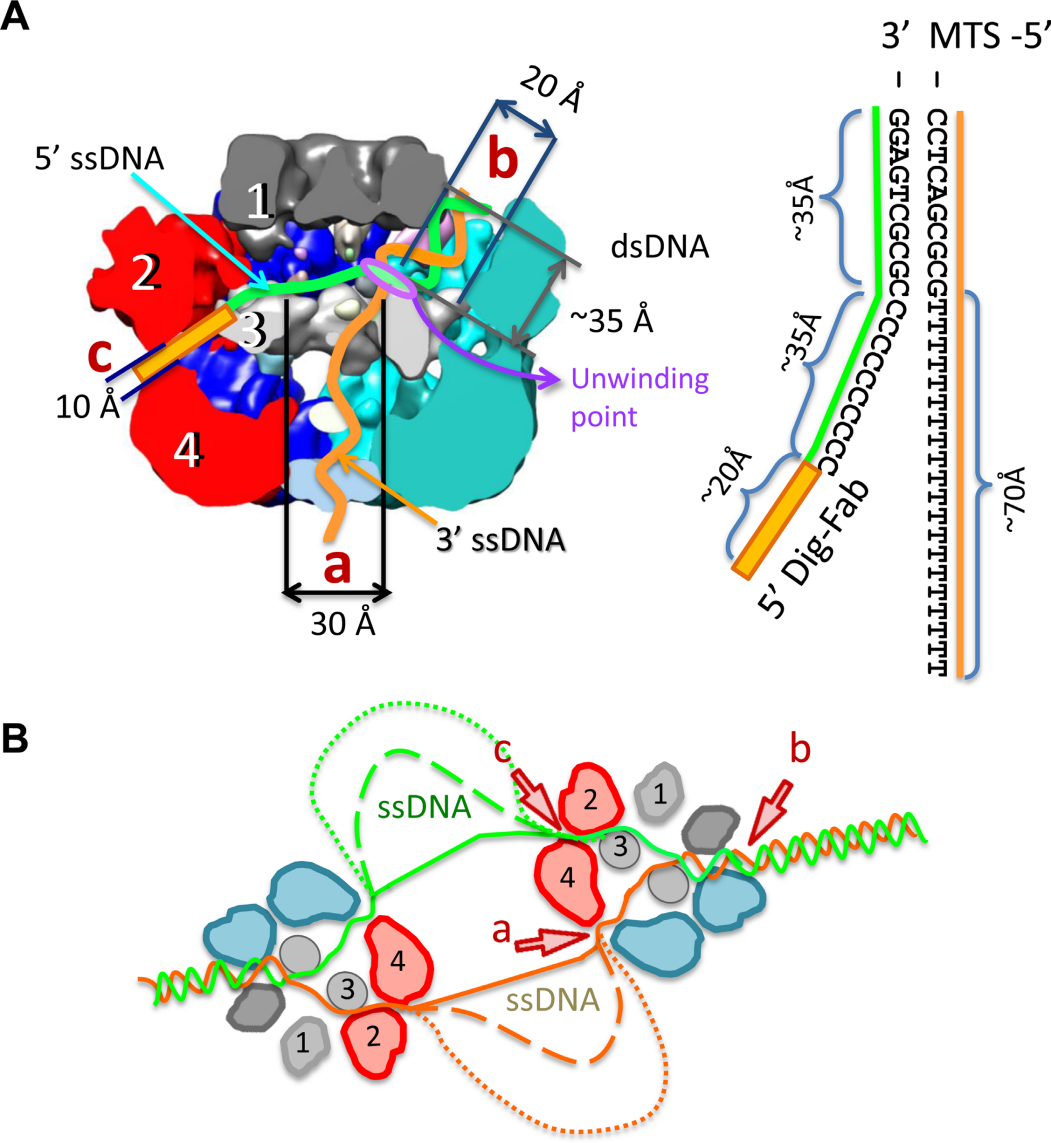
Diagram of the E1 DNA unwinding machine. (**A**) The model for replication fork engagement is shown with the sizes of length and diameters of tunnels. Schematic representation of the DNA fork used in experiments is shown to the right. The DNA lengths are dsDNA ∼35 Å; 5′ ssDNA, ∼35 Å and 3′ ssDNA, ∼70 Å. The lengths of ssDNA are based on corresponding lengths in duplex DNA and therefore could be larger. The length of the link between the digoxigenin group and DNA is also indicated (∼20 Å). It is proposed that dsDNA enters into the hexameric assembly through tunnel ‘b’ where it faces domain 3 (the ‘wedge’) and base pairs are separated at the entrance into the HD. The ‘active’ ssDNA strand is pulled along the central tunnel (towards exit ‘a’) by the ATP-fuelled HD motor. The ‘passive’ ssDNA strand exits via tunnel ‘c’. (**B**) Model of DNA unwinding by a double hexamer of E1. dsDNA is shown entering the E1 assembly as in (A) and the dashed lines indicate the accumulation of increasing amounts of ssDNA.

Significantly, the electron densities corresponding to the MTS and Fab labels are at fixed positions on opposing faces of the E1FL complex separated by a distance of ∼120 Å at an angle of ∼150° across the central axis of E1 (Figure 5B and C). The distance between the MTS and Fab labels (∼110 Å) measured across the protein complex is in accord with their length of separation in the fork construct (Figures 5C and 7). The best explanation for such an arrangement is that the dsDNA enters E1 so that the fork junction is above the helicase motor unit. Fab makes a contact with the E1 complex in the area of the second tier. It is clear that the two single stranded ends of the unwound DNA, exiting the E1 hexamer through tunnels ‘a’(3′) and ‘c’(5′) would be separated by a path of at least 200 Å around the outside of E1.

## DISCUSSION

The X-ray structure of the E1HD/ssDNA/ADP hexamer showed that the ‘active’ 3′ ssDNA strand, or leading replication strand, exits along the central axis of the HD (7). The currently favoured ‘steric exclusion’ model of DNA unwinding for E1 (20), while incorporating 3′ exit at the end of a central tunnel, is based on exclusion of dsDNA and the 5′ ssDNA strand outside the helicase at the opposite end of the tunnel and similar steric exclusion models are proposed for other hexameric helicases (9,18,21–23,37,38). However, the steric exclusion model is based on indirect observation and until now has not been validated with structural information on complexes with complete replication forks containing dsDNA as well as two single-stranded segments. One possible reason for this is that these replication complexes are highly dynamic, mobile and demonstrate conformational diversity, so complicating detailed structural analysis.

Here, we produced stable hexameric complexes of full length E1 with ssDNA and intact replication forks bound, determined structures at 18–23 Å resolution and localized DNA replication fork entrance and exit points at the hexamer surface by direct visualization of DNA ends labelled with MTS and Fab. The atomic structures of the E1 helicase domain determined without (17) and with ssDNA and nucleotide cofactor bound (7) are virtually identical with the C-α r.m.s. deviation of 0.35 Å. In these crystal structures, the E1 OD (residues 308–378) is highly symmetrical, while the ATP binding sites are found at the interfaces between adjacent AAA+ domains (residues 379–578) that show positional variations of up to 7.5 Å. It is possible therefore that the helicase domain could impose asymmetry on the N-terminal portion of the molecule mediated through the AAA+ domain positional asymmetry, but it is unlikely that protein–nucleic acid interactions are significantly different within the N-terminal half (residues 1–307) of the complex, with and without nucleotide cofactor bound. Together with DNA footprinting experiments, our structural observations indicate, first, that at least 10 bp of dsDNA enters inside the intact helicase complex and that unwinding most likely takes place at the entrance to the helicase domain (Figure 7). Second, labelling with MTS suggests, unexpectedly, that dsDNA enters not along the central tunnel but through the side tunnel ‘b’. We favour this interpretation because we observe clear density linking the MTS to the entrance of tunnel ‘b’, while the entrance to tunnel ‘a’ is unobstructed and there is a complete absence of density between E1 entrance ‘a’ and MTS in our models. However, we acknowledge that higher resolution data would be required to completely exclude an axial path for dsDNA entry. Third, labelling of the 5′ ssDNA end with Fab indicates that the lagging (5′ passive) strand exits the E1 complex on an opposing side to dsDNA entry via the narrower tunnel ‘c’. The negative stain EM benefits from excellent contrast, that is important for initial analysis of dynamic systems (39) and the resolutions obtained are more than sufficient for deducing DNA end-label locations (MTS and Fab) on the molecular surface of E1, that are separated by at least 200 Å.

Our observations therefore suggest a revision of the steric exclusion model proposed for E1 (20) where, alternatively, DNA strands are wedged apart within the hexameric assembly and not outside. The E1 helicase domain (residues ∼300–608) forms stable hexamers (12) with the active ssDNA strand bound in the central tunnel (7) and these complexes can unwind helicase substrates (12). In the absence of the N-terminal half of the protein (domains 1 and 2), and therefore the absence of additional channels that could accommodate DNA, these assemblies are likely to be operating by a strict steric exclusion mechanism. As such, the entrance to the helicase domain acts to wedge DNA strands apart while the passive ssDNA strand is excluded from the HD complex. Together with previous data locating the active ssDNA strand (7), our new structural data show that all DNA replication fork arms in the full length E1 hexamer are entering or leaving via well-defined openings that may be fixed for the duration of translocation. Thus, our model envisions the same wedging mechanism at that entrance to the helicase domain (Figure 7), but with all arms of the replication fork passing through discrete conduits. This could modify our understanding of the mechanism of DNA unwinding, since direct interactions with the dsDNA ahead of the fork and also the passive ssDNA strand could influence the efficiency of the unwinding process (2). The anatomy of E1-RFJ DNA engagement deduced suggests that the E1 hexamer may be operating like the heterotrimeric *E. coli* RecBCD helicase whose structure has also been determined in complex with a fork-like DNA molecule (40). In RecBCD, several DNA base pairs enter a short tunnel formed between the RecB and C subunit and an ‘arm’ of the RecB subunit contacts dsDNA 12 bp from the fork junction. DNA strands are split across a protein ‘pin’ provided by RecC, as the ssDNA tails are pulled through discrete channels by the RecB and D motors. It is likely that a specific protein segment corresponding to a ‘pin’ is involved in wedging DNA strands apart at the entrance to the E1 helicase domain.

It is unclear whether other hexameric helicases unwind dsDNA as proposed for E1. However, there are biochemical data widely supporting a steric exclusion model and structural data indicating multiple channels for DNA entry and exit in other hexameric helicases including T7 gp4 (37), bacterial DnaB (18), SV40 LTag (38), archaeal MCM (22,41) and the eukaryotic MCM2–7 complexes (42,43). Such data are not incompatible with our model incorporating inclusion of the RFJ within the hexamer, now proposed on the basis of the structural observations of E1FL–RFJ complex. Failure of these replicative hexameric helicases to displace a biotin-mediated streptavidin ‘roadblock’ on the passive ssDNA strand is frequently taken as evidence of its steric exclusion (e.g. refs. 20 and 38). However, SV40 L-Tag hexamers have been shown to bypass a covalently linked bulky adduct even on the active translocation strand, even though it is pulled through the motor domain of the toroid (38). A ring-opening mechanism was suggested, akin to that previously demonstrated in T7 gp4 (44). Given the similarities between E1 and L-Tag it is likely that the hexameric E1 protein ring can also open in a dynamic engagement with the RFJ. Notably, opening of only the upper N-terminal ring would be needed to by-pass obstacles such as DNA secondary structure or bound proteins on the lagging (passive) DNA replication strand. Indeed, the distortions observed in the protein-tagged RFJ complexes (Figure 5) in close proximity to streptavidin and Fab in particular which is submerged into the complex, not only demonstrate that the bound replication fork is intact but indicate that N-terminal domains 1 and 2 (∼residues 1–300) are flexible. Two independent rings (HD and the N-terminal-OBD ring in the case of E1), each engaging DNA and that can each open and close, could facilitate by-pass while ensuring that the helicase remains stably associated with its substrate.

In a toroidal protein–DNA complex the nucleic acid can be internal and completely surrounded by protein or lie on its surface. In the latter case only one face of a dsDNA helix would contact protein leaving the outer surface susceptible to nuclease cleavage. In OH• footprinting experiments such interactions produce very characteristic periodic cutting pattern progressing through protection to no protection with centres spaced 10–11 bases apart (one helical turn), as illustrated by lambda repressor and Cro proteins (34). The densitometry traces of E1FL–RFJ complexes (Figure 6A) show no evidence of periodicity but instead a uniform protection approaching two turns of the dsDNA helix. This is best interpreted as E1 completely encircling the DNA, forming a ‘sheath’. Furthermore, since we only observe hexamers of E1FL (Figure 2) or E1HD (12) in the presence of DNA it is unlikely that the observed protections are representative of partial E1–DNA complex formation. A corollary to this and consistent with our structural observations is that the RFJ is inside the complex in a chamber at the entrance to the helicase motor domain. The complete protection observed over the first five bases of the 5′ passive ssDNA are also consistent with their inclusion within the complex and exit most likely via a side tunnel ‘c’. Likewise, the DNA protection pattern for the 3′ ‘active’ strand is in agreement with its known passage through the central tunnel of the helicase motor domain bearing the ssDNA binding sites (7,24). Direct visualization of the paths taken by the replication fork strands will ultimately require higher resolution structures of intact E1–RFJ complexes that have thus far proven difficult to obtain for this class of helicase and, indeed, small asymmetric structures in general that do not lend themselves readily to cryo-EM (39,45,46). However, our structural data identifying the positions of the DNA ends with surface labels are in accord with the arrangement and dimensions of the tunnels in E1 with respect to the proposed path of the occupying DNA and the DNA protections we observe in footprinting experiments. Furthermore, our data do not exclude the possibility that an extended part of the lagging strand ssDNA is wrapped on the outer surface of E1, as in *Sulfolobus solfataricus* MCM, to enhance the unwinding process (22).

Our results, defining the structural arrangement of an intact E1 helicase complex and its orientation with respect to the RFJ, are consistent with recent FRET data that suggest the RFJ is located nearer the OBD and away from the HD (20). Furthermore, while we do not consider the continuity of the unwinding process here, our proposition that the fork junction is occluded within the E1 hexamer could explain the unevenness of unwinding of dsDNA observed for E1HD, which is ameliorated by the presence of the OBD domain (20), and is likely to be unperturbed in the case of full length E1 at the RFJ *in vivo*. Our model for dsDNA unwinding shown in Figure 7B also incorporates two E1 helicases acting in unison, consistent with the assembly of double hexamers at the origin of DNA replication (47).

The helicase-catalyzed DNA processing events in DNA replication are conserved and, like E1 (47), highly dynamic particles of *Drosophila melanogaster* and yeast MCM complexes, that incorporate ring opening and closing mechanisms, have also been observed during establishment of bi-directional DNA replication (39,48–51). Observations in archaeal systems also indicate that DNA can take alternative paths in associated hexameric MCM complexes, exemplified by the wrapping of dsDNA around the external surface of the putative *Methanothermobacter thermoautotrophicus* MCM pre-replication complexes (52). The new insights into E1 and how it engages the RFJ presented here therefore have broader implications for understanding how DNA processing machines work.

## ACCESSION NUMBERS

The EM density maps have been deposited in the EMDB with accession codes EMD-3087 for E1HD. EMD-3088, for E1FL asymmetrical structure, EMD-3089 for the E1FL symmetrical structure, EMD-3090 for the complex E1FL-MTS and EMD-3091 for the complex E1-MTS-FAB.

## SUPPLEMENTARY DATA

Supplementary Data are available at NAR Online.

SUPPLEMENTARY DATA
