# Supplementary material for: Structural basis for DNA strand separation by a hexameric replicative helicase
Source: Nucleic Acids Res. 2015 Aug 3;43(17):8551–63. doi: 10.1093/nar/gkv778 (PMC4787811; doi:10.1093/nar/gkv778)
Supplement: SUPPLEMENTARY DATA [file supp_43_17_8551__index.html]

Structural basis for DNA strand separation by a hexameric replicative helicase — Structural basis for DNA strand separation by a hexameric replicative helicase — Structural basis for DNA strand separation by a hexameric replicative helicase — SUPPLEMENTARY DATA 

# Structural basis for DNA strand separation by a hexameric replicative helicase

## SUPPLEMENTARY DATA

- SUPPLEMENTARY DATA
- SUPPLEMENTARY DATA
