# Supplementary material for: Structural basis for DNA strand separation by a hexameric replicative helicase
Source: Nucleic Acids Res. 2015 Aug 3;43(17):8551–63. doi: 10.1093/nar/gkv778 (PMC4787811; doi:10.1093/nar/gkv778)
Supplement: SUPPLEMENTARY DATA [file supp_gkv778_nar-01451-h-2015-File003.pdf]

Structural basis for DNA strand separation by a hexameric replicative helicase

Yuriy Chaban, Jonathan A. Stead, Ksenia Ryzhenkova, Fiona Whelan,

Katerina Lamber, Alfred Antson, Cyril M. Sanders, Elena V. Orlova

Supplemental information

Procedures

Protein expression, purification and DNA complex preparation. Full length BPV-1 E1 protein (E1FL) was expressed as a GST fusion, cleaved with thrombin and purified free from the tag by cation and anion exchange chromatography [24]. E1HD, residues 299-605, was expressed without a fusion tag and purified as described [12]. Antibodies (rabbit) were raised against the purified N-terminal residues 1-308 of E1 and purified using a polypeptide of E1 residues 1-129 covalently coupled to sepharose. HA epitope-tagged FLE1 was produced by insertion of a sequence encoding residues GGYPYDVPDYAG (HA epitope underlined) after residue 226, and purified as for E1FL. The corresponding epitope-tagged E1 retained full biochemical activity compared to the untagged protein and bound the anti-HA monoclonal antibody 12CA5 used against the epitope. The subunits for monovalent, tetrameric, streptavidin (MTS) were expressed from plasmids pET21a-Streptavidin-Dead and pET21a-Streptavidin-Alive, which were supplied from Dr. Alice Ting's Laboratory, MIT, via Addgene.org. The complex was prepared as described previously [25].

Protein-DNA complexes for electron microscopy were prepared by incubating protein (2-3 $mgml^{-1}$) and DNA at a 6:1 molar ratio before size exclusion chromatography (SEC) on a Superdex-200 HR 10/300 GL (GE Healthcare) gel filtration column (10 mM Tris-Cl pH 8.0, 225 mM NaCl, 5% v/v glycerol, 2 mM DTT, 0.1 mM PMSF, 0.1 mM EDTA). For the tagged RFL DNA complexes, monovalent, tetrameric streptavidin (MTS) or anti-digoxigenin Fab (Roche) were added after hexamer formation at a 1.5 molar excess over DNA and incubated for a further 10 minutes before chromatography (**Figure S3**). To analyse the HA epitope tagged E1FL-RFJ complex, antibody bound E1 hexamers were also purified by gel filtration. All E1 hexameric peak fractions were analysed by SDS-PAGE, pooled and dialysed against 500 volumes of SEC buffer without

glycerol prior to concentration. The DNA used for the E1HD complex was a partially single- and double-stranded RFJ-like molecule formed by annealing a single HPLC purified oligonucleotide, 5'- $(T)_{10}$GCGCGCGCGC$(T)_4$GCGCGCGCGC$(T)_{15}$. The E1FL complex was assembled on a $(T)_{30}$ oligonucleotide, or with RFJ like substrates formed by annealing the following oligonucleotides 5′-CCTCAGCGCGTTTTTTTTTTTTTTTTTTT and 3′-GGAGTCGCGCCCCCCCCCC, synthesized with or without 5′-biotin or digoxigenin for tagging.

DNA binding reactions, hydroxyl radical footprinting. DNA binding reactions were performed as described previously [12]. Oligonucleotides were 5′-end labelled with polynucleotide kinase and [γ^{32}P]-ATP (7000 Ci/mmol) or 3′-end using [α^{32}P]-dCTP and Klenow exo$^-$ (NEB). The sequence of the top strand (5′ end-labelled) was GGCTTGTATTTCACACCGCACCTCAGCGCGTTTTTTTTTTTTTTTTTTT and the bottom strand CCCCCCCCCGCGCTGAGGTGCGGTGTGAAATACAAGCC (annealed to the 5′ end-labelled top strand) or CCCCCCCCCGCGCTGAGGTGCGGTGTGAAATACAAG when 3′ end labelling the bottom strand. Both labelled/annealed substrates were gel purified before use. The binding buffer for complex formation was 20 mM Na phosphate pH 7.2, 135 mM NaCl, 0.1 % NP40, 0.1 mgml^{-1} BSA, 1 mM PMSF, 1 mM DTT. Binding reactions (50 µl) were assembled, with 0.5-2 µM E1 proteins and approximately a 6:1 ratio of protein to DNA. Reactions were incubated at room temperature for 20 minutes before addition of a 10-fold excess of unlabelled T18 competitor oligonucleotide and an additional 10 minutes' incubation. Hydroxyl radical footprinting was then performed according to the general guidelines of Dixon *et al.*, 1991 [26]. Reactions were extracted twice with phenol/chloroform and finally diluted with an equal volume of formamide (98%) loading dye for analysis on 15% urea-polyacrylamide sequencing gels. Analysis of dried gels was performed by phosphorimaging.

DNA unwinding reactions. Helicase assays were performed in 20 mM HEPES-NaOH pH 7.2, 135 mM NaCl, 0.1 % NP40, 0.1 mgml^{-1} BSA, 1 mM PMSF, 1 mM DTT, 3 mM $MgCl_2$ and 1 mM ATP. Reactions were incubated at 20°C for 60 minutes and processed as described in [12]. The substrates used were identical in sequence to that used OH• footprinting and without either the 5′ or 3′ ssDNA arms.

Electron microscopy, image processing and model fitting. All samples were applied on to carbon-coated copper grids (400 mesh, freshly glow-discharged in air) and stained with 2% uranyl acetate. Images were manually recorded on a Tecnai F20 electron microscope (FEI, Netherlands)

operated at 200 keV, using a Gatan Ultrascan 4000 4k × 4k CCD camera with an ultra-sensitive phosphor scintillator (Gatan, USA). Recording was done using a low dose mode (~20 electrons/Å^2) at a nominal magnification of 62,000 in a range of defocus settings (0.7-2.5 µk). All CCD frames were tested for image quality; ~ 5% were discarded due to poor quality resulting from astigmatism or drift (**Table S1**). Particle picking was carried out automatically using the program BOXER (EMAN2, [27]). Initial references were prepared using several manually selected protein complex images covering a number of views. Analysis of the CTF and correction was completed using the program CTFIT (EMAN2, [27]). The following image analysis was performed using IMAGIC-5 [28]: Images were normalized to the same standard deviation and band-pass filtered; the low-resolution cut-off was ~100 Å to remove uneven background in particle images and the high-resolution cut-off was ~7 Å. Images were then subjected to an alignment procedure followed by statistical analysis **(Figure S1**, **Table S1**). Alignment and classification of images was performed as previously described [28,29] and yielded classes representing characteristic views of the molecule. Angular orientations of class averages were determined by angular reconstitution (AR). 3D maps were calculated using the exact-filter back projection algorithm [29]. Structural analysis was performed using several starting models with several different sets of image classes for *ab initio* reconstructions.

Primary structural analysis for E1HD and E1FL complexes was performed using an *ab initio* approach where the orientations of the best 10-15 image classes were determined by AR without and then with symmetry constraints. The first reconstructions were used for the following rounds of alignment and classification of images. The structure of the E1HD complex was refined by an iterative procedure with the number of classes gradually increased with symmetry C6 imposed. The final symmetrical reconstruction of the E1HD was calculated from the best 450 classes. The C6 model of E1FL provided a 3D reconstruction with a high correlation (> 0.6) between re-projections and input classes. The final asymmetrical reconstruction of the E1FL complex was obtained with 5-7 images per class. The structures of both asymmetrical and symmetrised maps of the E1FL complex were refined by independent procedures using 500 classes in both cases. Resolution of the maps was assessed using the 0.5 threshold of Fourier Shell Correlation [29], which corresponds to 19 Å, 23 Å, and 18 Å respectively for E1FL (symmetrical), E1FL asymmetrical and E1HD, (symmetrical) (**Figures S2 and S4)**.

Ab-E1 contacts were visualised using negative stain EM. To analyse the E1/E1 anti-N-terminal antibody complex, ~8500 images of the complex with one or two antibodies bound were selected manually and subjected to the standard procedure of centring and classification [28]. The

best characteristic side views (~1000 images) with E1 and one antibody were selected from 150 classes and images constituting these classes were extracted into separate groups. The subsets of images were aligned and those with the highest correlation were averaged. Statistical analysis of the aligned images of ab-E1FL helped selection of side views of the complex where the three rings of the E1FL structure are clearly seen. Since one ring of the complex has a slightly bigger diameter and the HD domain always appears much denser in EM, the orientation of each complex was clear in averaged and aligned images. Similar procedures to those described above were used for the HA-epitope mapping experiment (**Figure S5 and S6)**.

A complex of full length E1 labelled with MTS on the dsDNA 5′ end and the 5′ ssDNA end with Fab was generated as described above and stained with 2% UA. We have used 260 frames of images and collected ~18000 particles from those frames. After MSA analysis 2000 classes were obtained. Representative class averages used for 3D reconstruction are shown in **Figure S1H.** However, during image processing it was found that the sample was heterogeneous having only a partial occupancy of Fab (~30% of particles). To solve this problem an *a posteriori* 3D statistical analysis was used [30]. Once the data were separated into two subsets, we determined two structures, one with two labels and another with only MTS attached to the dsDNA end.

Domain fitting into the 3D map of E1HD and E1FL was initially performed manually using PyMOL (http://pymol.sourceforge.net/) and verified by automated fitting with Chimera [31] and Veda [32] software. To identify positions of subunits and their domains we used the segmentation function in Chimera. Illustrations were generated using Chimera. Surface representations (unless stated otherwise) are displayed at a threshold level of 1σ (standard deviation of densities within EM maps) that corresponds to ~100% of the expected mass at the specific protein density of 0.84 kDa/$Å^3$ **(Figure 2**, **Table S2**).

SUPPLEMENTAL FIGURE LEGENDS

Supplemental Figure 1. Electron microscopy and image analysis of E1 complexes. EM images of **(A)** E1FL bound to ssDNA (T30), **(B)** E1HD bound to a RFJ DNA substrate, **(C)** E1FL bound to a RFJ substrate labelled on the 5′ end of the dsDNA strand with monovalent, tetrameric, streptavidin (MTS), and **(D)** RFJ-E1 complexes dual-labelled with MTS on the 5′ end of the dsDNA and anti-digoxigenin Fab on the 5′ ssDNA end. Images were taken at 62K magnification after staining with 2% uranyl acetate on carbon-coated copper grids. Representative classes are shown for (A) E1FL bound to ssDNA (T30) in **(E)**, for (B) E1HD bound to a RFJ DNA substrate in **(F)**, for (C) E1FL bound to a RFJ substrate labelled on the 5′ dsDNA strand with MTS in **(G)** and for (D), RFJ-E1 complexes dual-labelled with MTS on the 5′ dsDNA end and anti-digoxigenin Fab on the 5′ ssDNA end in (**H**).

Supplemental Figure 2. Orientation of the E1 complex by antibody binding and alignment of E1 structures. (**A**) Binding of antibodies (ab) specific to N-terminal residues 1-129. The upper row demonstrates side class averages of the E1 complex without ab. The second row shows side views with extra densities on the top of the wide ring. The third row shows the same images where the extra densities are highlighted. The last two bottom rows demonstrate the difference between images with ab and without ab, allowing localisation of the ab in the E1 complex and to identify positions of N-termini. **(B)** Top, surface views of the 3D EM reconstruction of E1HD. The left panel demonstrated fitting of X-ray (PDB 2V9P, [17]) model into the EM map. Below, the first row - orthogonal projections of the E1FL structure (FL), the next row shows the orthogonal projections of the E1HD reconstruction (HD). The bottom row represents the differences between two sets of re-projections demonstrating extra density located on the top of the helicase domain. **(C)** Distribution of angles for the dual-labelled RFJ-E1 complexes.

Supplemental Figure 3. Assembly and purification of FLE1 complexes with tagged RFJ DNA. (A) Substrates used in this study. **(B)** Method work-flow for complex assembly. **(C)** Chromatography trace (Superdex S200) and **(D)** SDS-PAGE gel analysis for the E1FL/RFJ/MTS/Fab complex.

Supplemental Figure 4. Fourier shell correlations for all structures. Resolution of the maps was assessed using the 0.5 threshold of Fourier Shell Correlation, which is 19 Å for E1HD, 18 Å for E1FL, 18 Å for E1FL+MTS and 20 Å for E1FL+MTS+Fab (**Table S1).**

Supplemental Figure 5. Internal epitope tagging of the E1 OBD. The E1 OBD structure (residues 159-303, (1KSX, [33]) is shown with secondary structural elements colored red, helix; yellow, sheet and green, loops. The residues E226 and G227 are shown as green spheres. A 12 residue peptide (GGYPYDVPDYAG) containing the haemaglutinin (HA) epitope (underlined) was inserted into a small exposed surface loop of the OBD, after residue 226. Recombinant epitope-tagged E1FL was purified for biochemical and structural analysis.

Supplemental Figure 6. Labelling of the E1 complex with antibody to the OBD. (A) Binding of antibodies specific to the HA-epitope tagged OBD (**Figure S5**). The upper row demonstrates side view class averages of the E1 complex with ab. The bottom row shows the same views with ab and E1 outlined in orange and blue ovals correspondingly. The right panel shows the images of E1 where no labels are observed. **(B)** Model representation of the E1FL complex (EM structure) bound to an antibody (ab) (surface of the atomic structure, pdb code 1IGJ). E1FL is shown in cyan, the ab is in light blue. Orientations are approximately the same as in upper left panel in (A). **(C)** The upper rows represent the difference between images in the left upper row in (A) and the first class average D shown on the left panel. The upper row of the right panel shows the differences between the upper left row in (A) and the second class average E on the left upper row (A). In the bottom rows positions of ab are highlighted with orange ovals.

SUPPLEMENTAL TABLES

Supplemental Table 1. Information on data collected and processed.

Sample	# CCD frames used	# particles selected	# classes used in final results	Resolution (Å)
E1HD	30	8265	450	19
E1FL asym	45	3553	500	23
E1FL C6	45	3500	500	18
E1FL+AB	37	1040 side views	25	n/a
E1FL+MTS	210	24000	750	18
E1FL+MTS+Fab	260	18000	750	20

Supplemental Table 2. Results of rigid body fitting of domains into EM maps. The best results are shown in bold, while the second best fittings that differ by some shifts and rotations are shown in light grey.

**Domain**	**PDB**	**CCC**									
		**E1HD**		**E1FL-C6**		**E1FL-C1**		**E1FL+RFJ +MTS**		**E1FL+RJF +MTS + FAB**	
		Best	Second best	Best	Second best	Best	Second best	Best	Second best	Best	Second best
FAB	1IGJ	-	-	-	-	-	-	-	-	**0.73**	0.71
MTS	3RY1	-	-	-	-	-	-	**0.80**	0.74	**0.89**	0.87
Helicase domain	2V9P	**0.66**	0.64	**0.72**	0.71	**0.66**	0.65	**0.80**	0.78	**0.86**	0.85
dsDNA	model	-	-	-	-	**0.63**	0.61	**0.79**	0.7	**0.89**	0.88
OBD chG	1KSX	-	-	**0.79**	0.77	**0.87**	0.86	**0.81**	0.74	**0.85**	0.82
OBD chH		-	-	**0.81**	0.80	**0.85**	0.84	**0.82**	0.81	**0.82**	0.80
OBD chI		-	-	**0.80**	0.78	**0.85**	0.84	**0.77**	0.76	**0.83**	0.81
OBD chJ		-	-	**0.78**	0.77	**0.85**	0.84	**0.79**	0.78	**0.88**	0.85
OBD chK		-	-	**0.76**	0.74	**0.82**	0.80	**0.80**	0.78	**0.83**	0.81
OBD chL		-	-	**0.76**	0.75	**0.85**	0.83	**0.74**	0.73	**0.90**	0.87

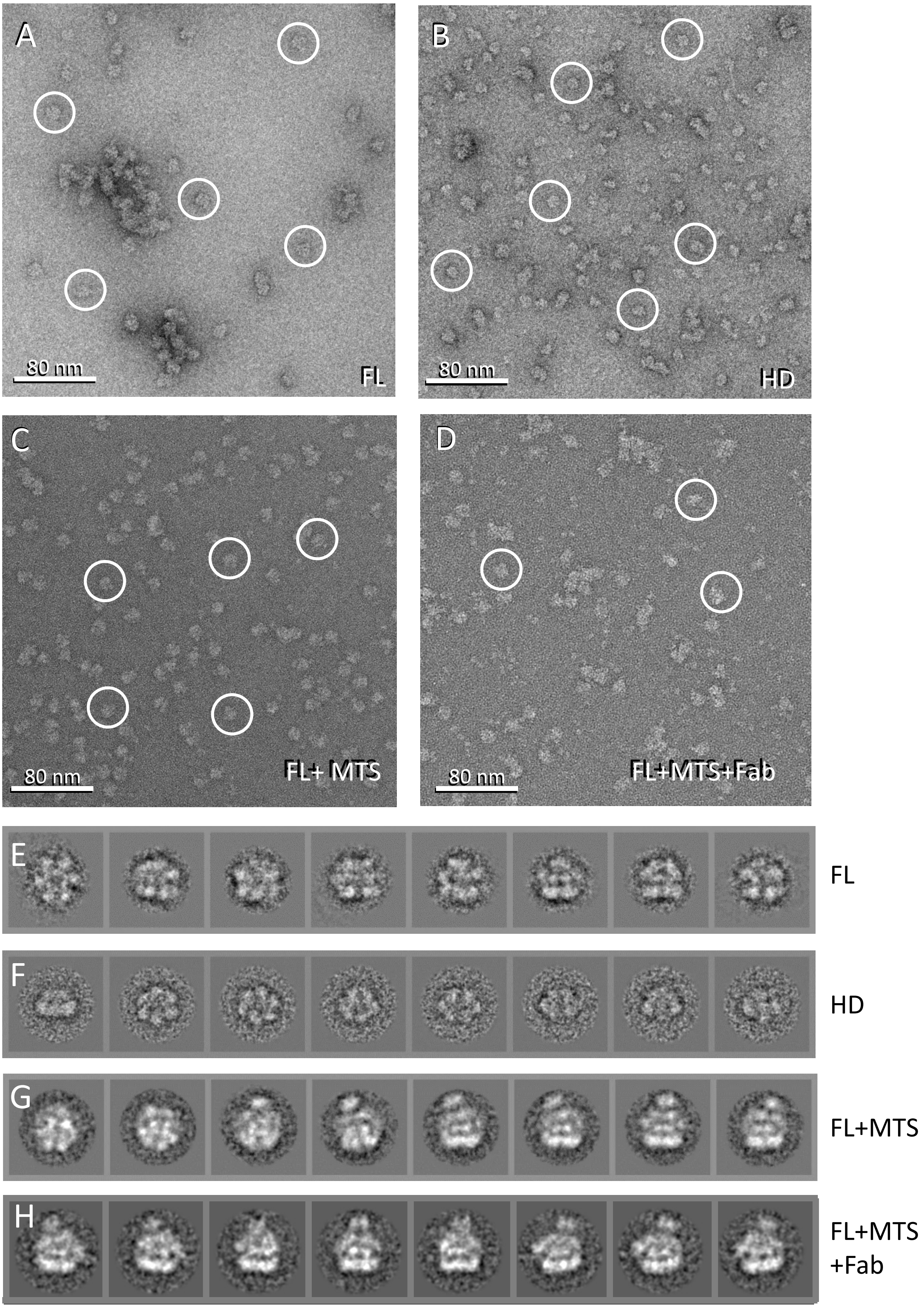

Supplemental Figure 1

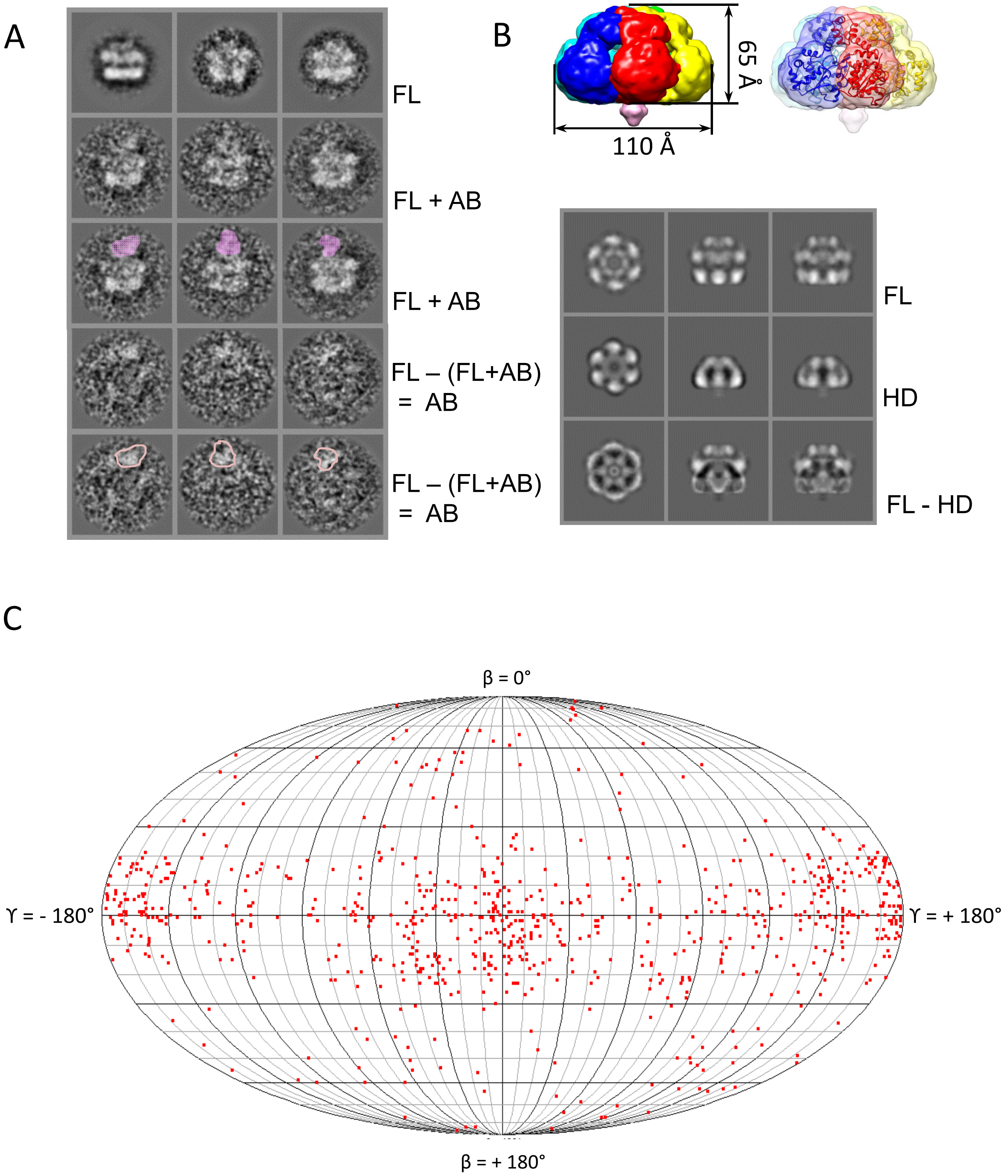

Supplemental Figure 2

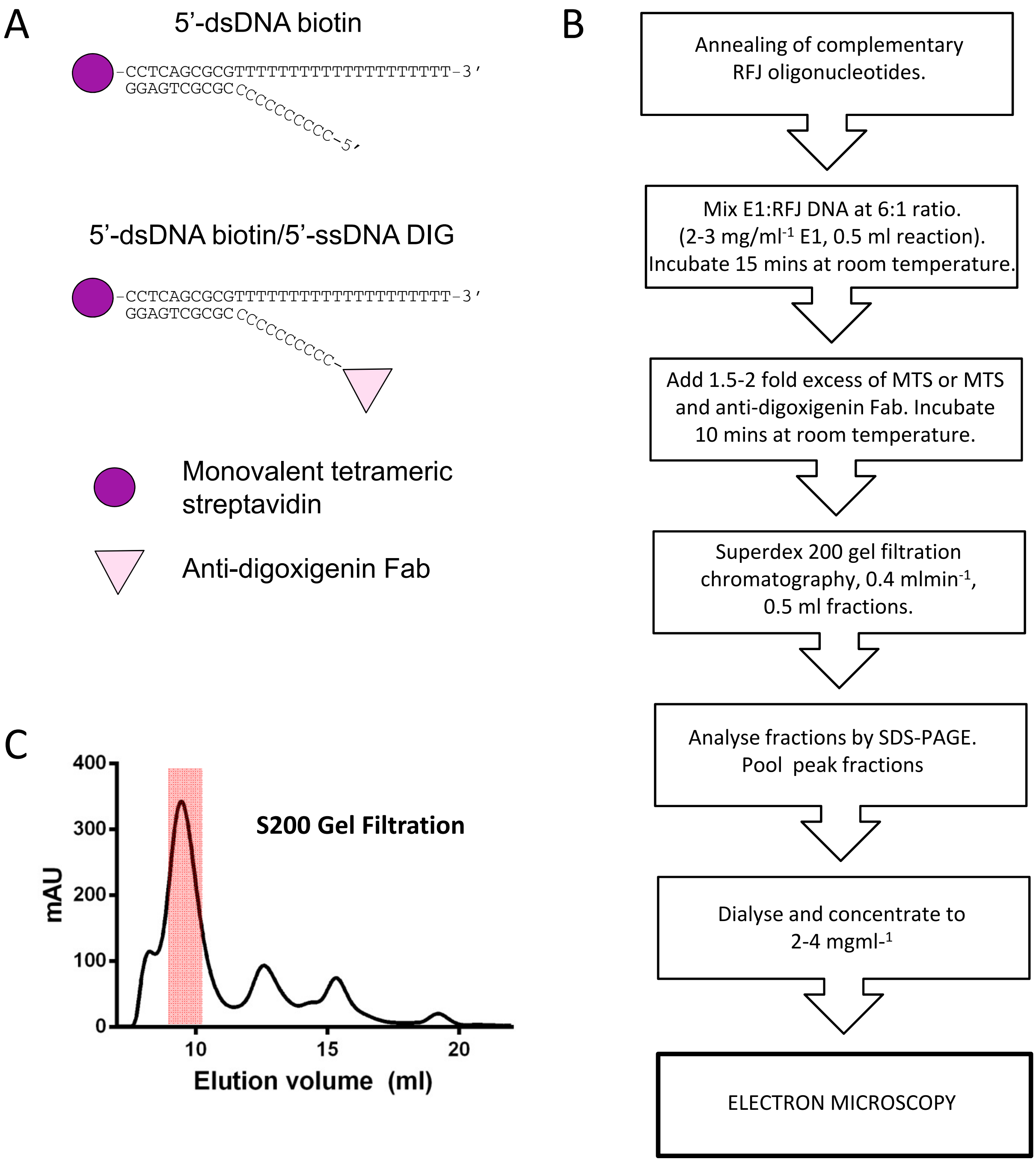

Elution volumes:
Fab, 16.3 ml
MTS, 15.3
RFJ, 15 ml
E1 monomer, 12.5 ml
RFJ+MTS, 13.4 ml
RFJ+Fab, 13.6 ml
RFJ+MTS+Fab, 12.4 ml
E1/RFJ/MTS, 9.5 ml
E1/RFJ/Fab, 9.64 ml
E1/RFJ/MTS/Fab complex, 9.85 ml

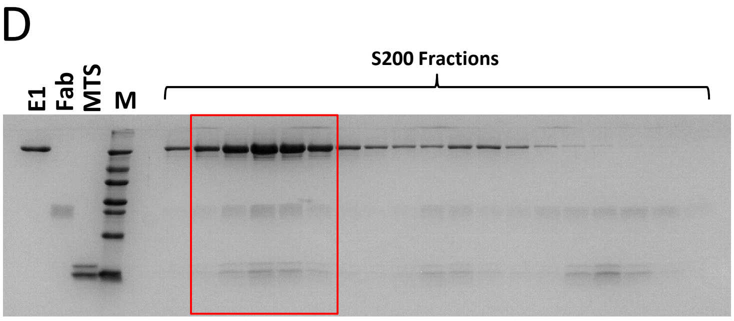

Supplemental Figure 3

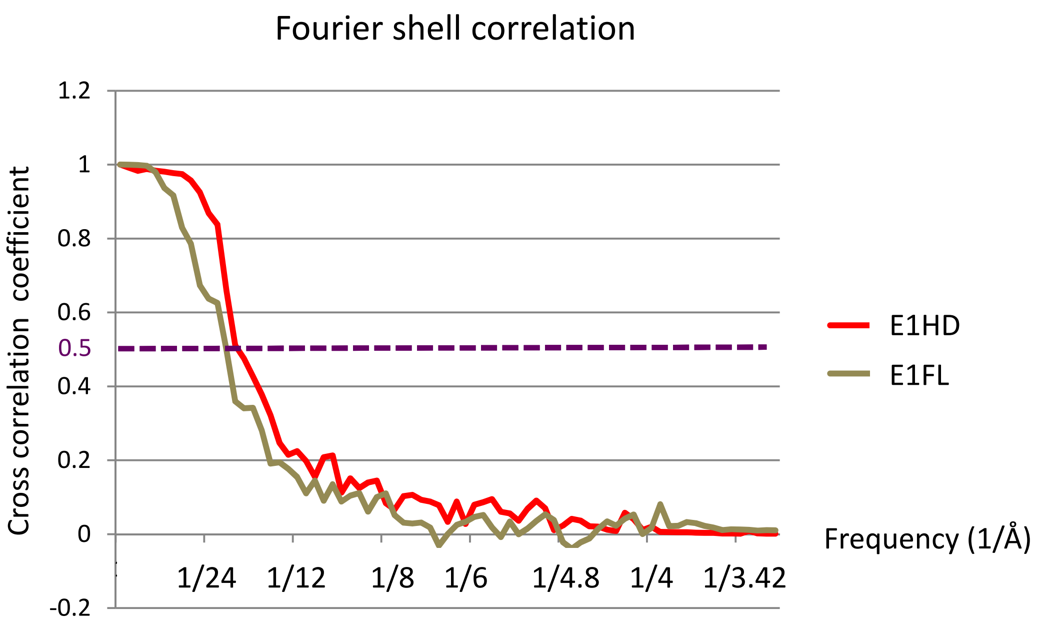

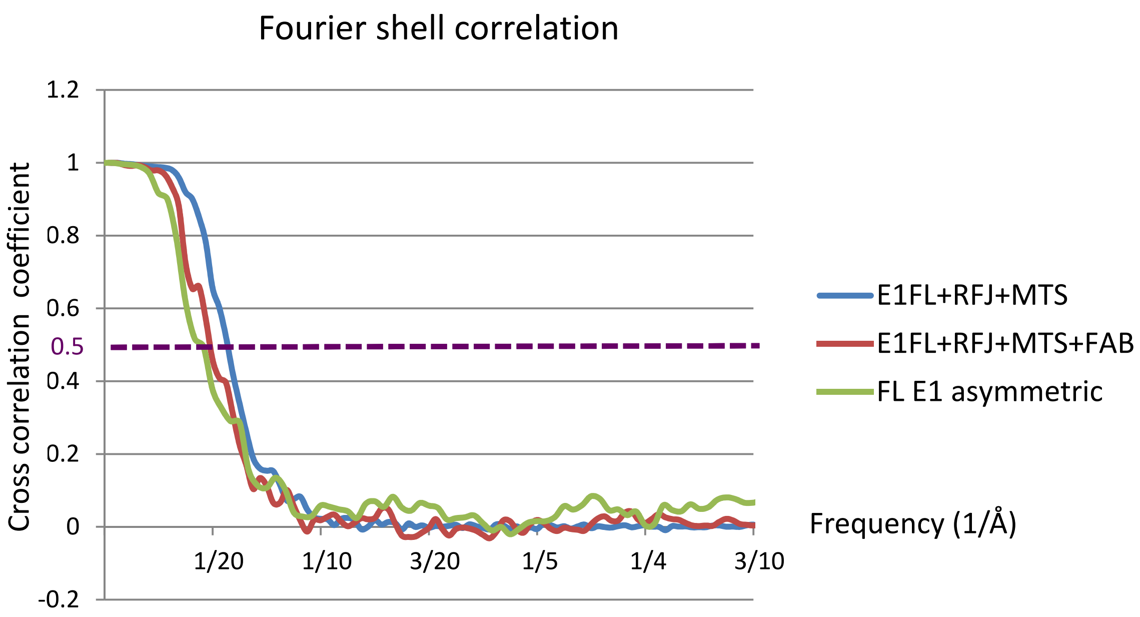

Supplemental Figure 4

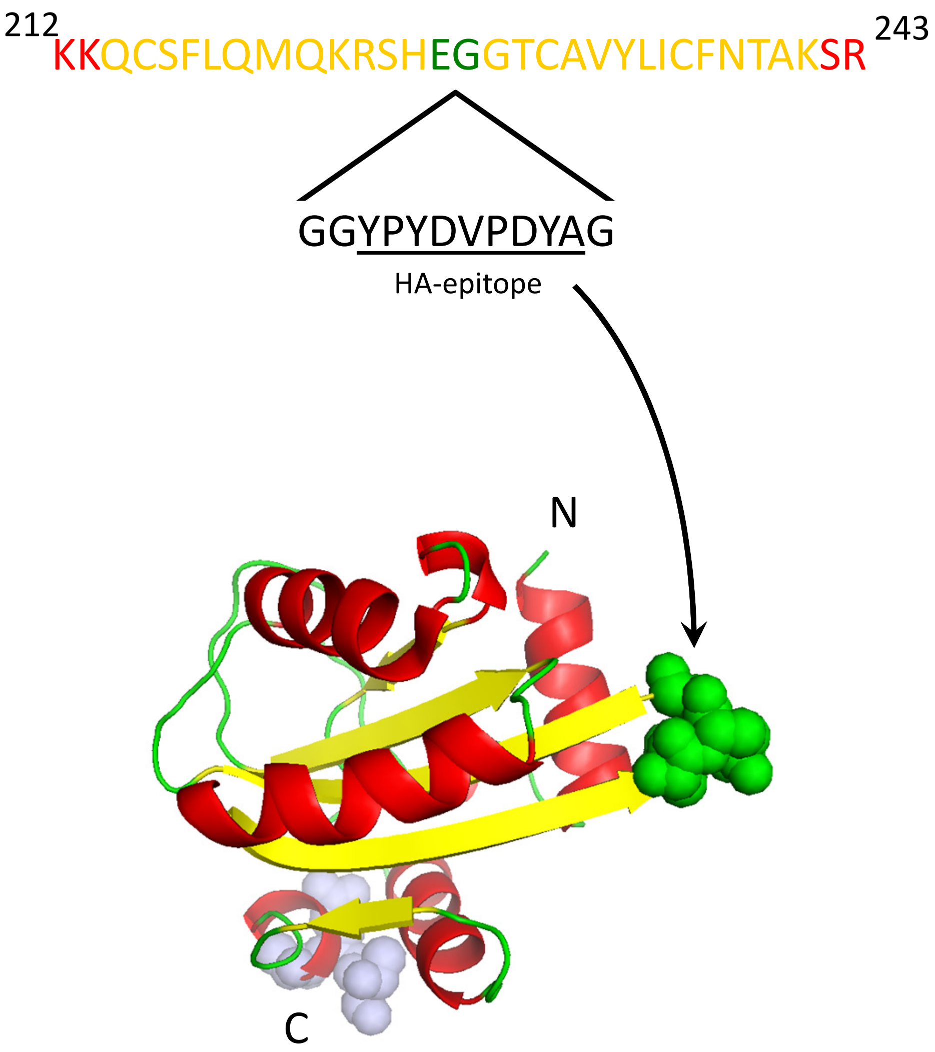

Supplemental Figure 5

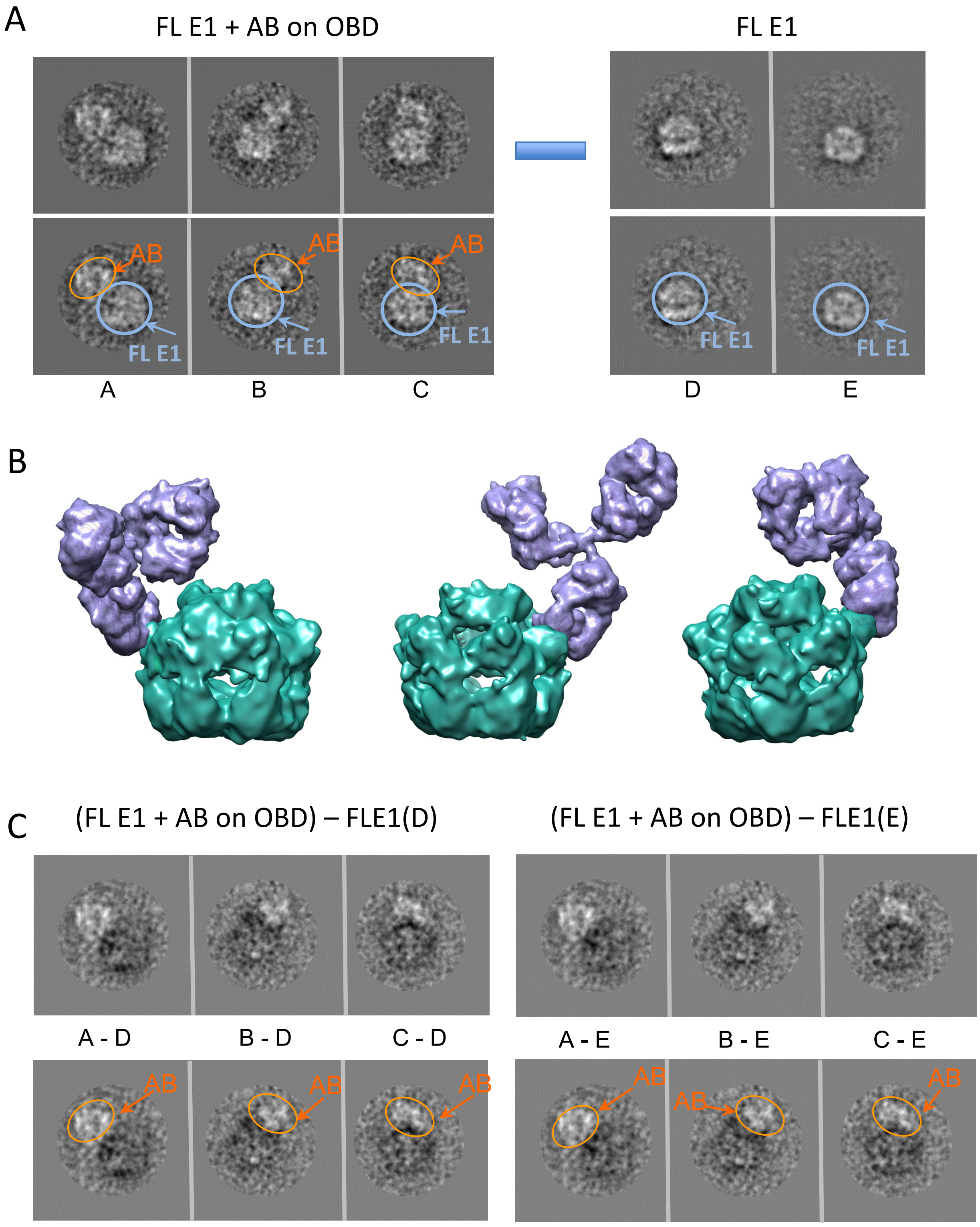

Supplemental Figure 6
